# Ipconazole Induces Oxidative Stress, Cell Death, and Proinflammation in SH-SY5Y Cells

**DOI:** 10.3390/toxics11070566

**Published:** 2023-06-30

**Authors:** Carlos Villaorduña, Mariano Mendoza-Carlos, Manuel Chuyma, Jhon Avilés, Ayda Avalos-Diaz, Ronald Lozano-Reategui, Juan Garcia-Ruiz, Nadia Panduro-Tenazoa, Jessy Vargas, Ysabel Moran-Quintanilla, José-Luis Rodríguez

**Affiliations:** 1 Faculty of Pharmacy, Universidad Nacional Mayor de San Marcos, Lima 15021, Peru; 2 Agroforestry Department, Universidad Nacional Intercultural de la Amazonia, Pucallpa 25004, Peruaavalosd@unia.edu.pe (A.A.-D.);; 3 Faculty of Veterinary, Universidad Complutense de Madrid, 28040 Madrid, Spain

**Keywords:** ipconazole, oxidative stress, cell death, neurotoxicity

## Abstract

Ipconazole is an antifungal agrochemical widely used in agriculture against seed diseases of rice, vegetables, and other crops; due to its easy accumulation in the environment, it poses a hazard to human, animal, and environmental health. Therefore, we investigated the cytotoxic effect of ipconazole on SH-SY5Y neuroblastoma cells using cell viability tests (MTT), ROS production, caspase3/7 activity, and molecular assays of the biomarkers of cell death (Bax, Casp3, APAF1, BNIP3, and Bcl2); inflammasome (NLRP3, Casp1, and IL1β); inflammation (NFκB, TNFα, and IL6); and antioxidants (NRF2, SOD, and GPx). SH-SY5Y cells were exposed to ipconazole (1, 5, 10, 20, 50, and 100 µM) for 24 h. The ipconazole, in a dose-dependent manner, reduced cell viability and produced an IC_50_ of 32.3 µM; it also produced an increase in ROS production and caspase3/7 enzyme activity in SH-SY5Y cells. In addition, ipconazole at 50 µM induced an overexpression of Bax, Casp3, APAF1, and BNIP3 (cell death genes); NLRP3, Casp1, and IL1B (inflammasome complex genes); and NFκB, TNFα, and IL6 (inflammation genes); it also reduced the expression of NRF2, SOD, and GPx (antioxidant genes). Our results show that ipconazole produces cytotoxic effects by reducing cell viability, generating oxidative stress, and inducing cell death in SH-SY5Y cells, so ipconazole exposure should be considered as a factor in the presentation of neurotoxicity or neurodegeneration.

## 1. Introduction

Pesticides are substances used as insecticides, fungicides, herbicides, rodenticides, molluscicides, and nematicides, and their role in agricultural development is very important because it optimizes production and reduces economic losses [1]. Due to the high demand for pesticides, their production has increased in recent decades, along with their abuse and indiscriminate use [2], which have caused pesticides to contaminate the environment and have negative impacts on human health [3].

Fungicides are chemical pesticides widely used in agriculture to reduce crop infestation by pathogenic fungi that reduce crops [4]. Despite this, it is believed that these compounds could have negative effects on the growth, photosynthesis, and other physiological activities of plants [4]. Triazole fungicides, widely used in agriculture, have also been reported to contaminate freshwater ecosystems. In fish, exposure to triazole fungicides has been observed to cause strong metabolic disturbances and alterations of metabolic parameters [5]. 

Triazole fungicides have a broad spectrum of action against pathogens by inhibiting the activity of lanosterol 14α-demethylase, a key enzyme for ergosterol biosynthesis in fungi [6]. The widespread use of triazoles has allowed their accumulation in the environment in substantial amounts, and they are causing adverse effects in non-target organisms, posing a great risk to environmental and human health [7]. It has been experimentally demonstrated that triazoles can reduce skeletal formation in developing zebrafish, promoting bone malformations [8]. In mammals, in an ex vivo study it was observed that the murine heart is sensitive to the triazole tebuconazole, which was able to induce cardiac arrhythmia and cardiac electrical remodeling by blocking calcium and potassium currents; both effects produced by tebuconazole represent a potential risk to mammalian cardiac function [9]. 

Ipconazole is a fungicide primarily for agricultural use that was developed in the 1980s and is widely used throughout the world [10]. The abuse of ipconazole and its negative effects on animal and human health are being studied [11]. However, there are few studies discussing the effect of ipconazole on altering health. In [12], the authors report only the presence of ipconazole residues in grains that could be factors in altering consumer health. In another study, zebrafish embryos treated with ipconazole at low doses exhibited cell death independent of the caspase enzyme, suggesting that ipconazole has the potential to alter neurodevelopment through dysregulation of mitochondrial homeostasis [13].

The present study evaluates the neurotoxicological impacts of the fungicide ipconazole on human neuroblastoma SH-SY5Y cells. This in vitro study was performed to determine the concentration-dependent cytotoxicity of ipconazole using the MTT assay, ROS generation, and caspase3/7 activity and to assess the expression of cell death (Bax, Casp3, APAF1, BNIP3, and Bcl-2); inflammasome complex (Casp1, NLRP3, and IL1β); proinflammatory (NFκB, TNFα, and IL6); and antioxidant (NRF2, SOD, and GPx) genes.

## 2. Materials and Methods

### 2.1. Reagents

Ipconazole (C_18_H_24_ClN_3_O) with a purity of 98.96% (*g*/*g*) was obtained from LGC group (Barcelona, Spain). 3-[4,5 dimethylthiazol-2-yl]-2,5-diphenyl-tetrazolium bromide (MTT), gentamicin, penicillin G and streptomycin, 2′,7′-dichlorofluorescin-diacetate (DCFH-DA), and Dulbecco’s phosphate-buffered saline (DPBS, D8537) were obtained from Sigma-Aldrich (Madrid, Spain). Dimethyl sulfoxide (DMSO) of analytical grade and all other usual laboratory reagents were acquired from Panreac (Barcelona, Spain). Nucleo-spin-RNA, qPCRBIO-cDNA-synthesis, ICgreen-amplification-PCR, DMEM-culture-media, and fetal bovine serum (FBS) were obtained from Cultek (Madrid, Spain). The Apo-ONE^®^ Homogeneous Caspase-3/7 assay kit was acquired from Promega (Madison, WI, USA). All other chemical reagents used were of high purity for cell and molecular biology and were available in the laboratory.

### 2.2. Cell Culture

Human SH-SY5Y cells (ATCC^®^ CRL-2266™) undifferentiated were obtained from American Type Culture Collection (ATCC, Manassas, VA, USA). The cells were cultured and passaged in DMEM with FBS (10%) and 50 mg/L of gentamicin, penicillin, and streptomycin. Cells were incubated in humidity with 5% CO_2_ and 95% air and at 37 °C; the culture medium was changed every other day.

Cells were treated with ipconazole (1, 5, 10, 20, 50, and 100 µM, dissolved in DMSO) in DMEM-F12 (without phenol red) with 1% FBS for 24 h. A vehicle control group (0.1% DMSO) was also used in each experiment, and to maintain the physiological conditions of the SH-SY5Y cells, they were used with fewer than 12 passages [14].

### 2.3. Cell Viability Evaluation (MTT)

The MTT assay was used to observe whether the mitochondria of viable cells are able to reduce the tetrazolium-MTT reagent to formazan [15]. Briefly, after ipconazole treatments, 0.5 mg/mL MTT was added as a final concentration to each well and incubated for 2 h at 37 °C and 5% CO_2_, this time allowing metabolically active SH-SY5Y cells to reduce tetrazolium-MTT (yellow color) to a formazan salt (purple color). After 2 h of incubation, the supernatant was removed from each well and 150 µL of DMSO was added to solubilize the formazan salt. The absorbance was measured at 540 nm (SPECTROstar BMG microplate reader). Cell viability is represented as % of control.

### 2.4. ROS Production

Oxidative stress can be assessed through the intracellular production of ROS according to standardized protocols using the DCFH-DA fluorescence assay [14]. DCFH-DA enters the cell and is hydrolyzed by esterases that allow the release of DCFH and its reaction with ROS to generate a detectable fluorescent compound. Briefly, after ipconazole treatments, 10 µM DCFH-DA was added to each well (2 × 10^5^ cells/well under incubation conditions) in a black multi-well plate for 30 min and immediately measured in a fluorescent microplate reader (FLx800 fluorimeter, BioTek, VT, USA) at 485 nm/530 nm (λ excitation/λ emission).

### 2.5. Apoptotic Assay with Caspase3/7 Activity

SH-SY5Y cells (15 × 10^3^ cells/well) were seeded in black 96-well plates. After ipconazole treatments, Apo-ONE^®^ Caspase3/7 was prepared and used according to the manufacturer’s instructions (fluorescent caspase3/7 substrate, rhodamine 110 Z-DEVD-R110, and bifunctional cell lysis buffer). The buffer lyses cultured SH-SY5Y cells, and the substrate and buffer were combined to make the Apo-ONE^®^ Caspase3/7 reagent that was added directly to each well. Rhodamine 110 emits fluorescence at 485/528 nm (λ excitation/λ emission), which was measured with the plate reader (FLx800, BioTek, VT, USA). The fluorescent product generated is an active caspase3/7 signal in cells. Data were evaluated as % of control [14].

### 2.6. Molecular Assay by Real-Time PCR

SH-SY5Y cells were seeded in 25 mL flasks in triplicate for each condition (control, vehicle, ipconazole 20 µM, and ipconazole 50 µM). After 24 h of the treatments, total RNA was obtained using NucleoSpin^®^-RNA-Plus (Macherey-Nagel, Düren, Germany, based on the use of silica columns) according to the manufacturer’s instructions. Total RNA was quantified using a nanospectrophotometer (Microdigital, Seoul, Republic of Korea), obtaining A260/A280 ratios close to 2.0 in all samples. The cDNA synthesis was obtained from 1 µg of total RNA by retrotranscription using the qPCRBIO cDNA synthesis kit (PCR Biosystems, PA, USA) according to the manufacturer’s instructions. Finally, the cDNA was diluted in nuclease-free water (*v*:*v*, 1:2) and stored at −80 °C. Real-time PCR (qPCR) assays to assess the expression of genes related to cell death (Bax, Casp3, APAF1, BNIP3, and Bcl2), inflammasome (NLRP3, Casp1, and IL1β), inflammation (NFκB, TNFα, and IL6), and antioxidant capacity (NRF2, SOD, and GPx) were performed with a real-time PCR system (BioRad CFX, CA, USA), using ICgreen Mastermix (Nippon Genetics, Duren, Germany) according to the manufacturer’s instructions. For RT-PCR, primers with 400 nM concentrations were used, and the thermocycling protocol was: 95 °C for 2 min and 40 cycles of 5 s at 95 °C and 30 s at 60 °C.

Bax (Bcl-2-associated X protein): ‘CCCCCGAGAGGTCTTTTTCC’ ‘CCTTGAGCACCAGTTTGCTG’
Casp3 (Caspase 3): ‘GTGGAGGCCGACTTCTTGTA’ ‘TTTCAGCATGGCACAAAGCG’
APAF1 (Apoptotic protease-activating factor 1): ‘TCTTCCAGTGGTAAAGATTCAGTT’ ‘CGGAGACGGTCTTTAGCCTC’
BNIP3 (BCL2-interacting protein 3): ‘CCTCAGCATGAGGAACACGA’ ‘GCCACCCCAGGATCTAACAG’
Bcl2 (B-cell lymphoma 2): ‘TCTCATGCCAAGGGGGAAAC’ ‘TCCCGGTTATCGTACCCTGT’
NLRP3 (NLR family pyrin domain containing 3): ‘CCCCGTAATCAACGGGACAA’ ‘AGCCAAATGCTTACCAGAAAGT’
Casp1 (Caspase 1): ‘GAAAAGCCATGGCCGACAAG’ ‘GCCCCTTTCGGAATAACGGA’
IL1β (Interleukin-1 beta): ‘CCAGCTACGAATCTCCGACC’ ‘TATCCTGTCCCTGGAGGTGG’
NFκB (Nuclear factor kappa B): ‘TTTTCGACTACGCGGTGACA’ ‘GTTACCCAAGCGGTCCAGAA’
TNFα (Tumor necrosis factor alpha): ‘CTGGAAAGGACACCATGAGCA’‘TCTCTCAGCTCCACGCCATT’
IL6 (Interleukin 6): ‘CCAGTACCCCCAGGAGAAGA’ ‘CAGCTCTGGCTTGTTCCTCA’
NRF2 (Nuclear factor erythroid 2-related factor 2): ‘CTGGTCATCGGAAAACCCCA’ ‘TCTGCAATTCTGAGCAGCCA’
SOD (Superoxide dismutase): ‘CCACTGCTGGGGATTGATGT’ ‘CGTGGTTTACTTTTTGCAAGCC’
GPx (Glutathione peroxidase): ’TTCGAGCCCAACTTCATGCT’ ‘CGATGTCAGGCTCGATGTCA’.
GAPDH (glyceraldehyde-3-phosphate dehydrogenase): (‘GAGAAGGCTGGGGCTCATTT‘ ‘AGTGATGGCATGGACTGTGG’) was used as a housekeeping gene. We extracted the efficiencies from the raw data using LinRegPCR software 20210614 [16].

### 2.7. Statistics

Data obtained from cell culture studies were analyzed using one-way ANOVA followed by Tukey’s post hoc test. Data were statistically significant at *p* < 0.05. GraphPad Prism version 8.0 was used for statistical analysis.

## 3. Results

### 3.1. Effect of Ipconazole on SH-SY5Y Cell Viability

**Figure 1:**
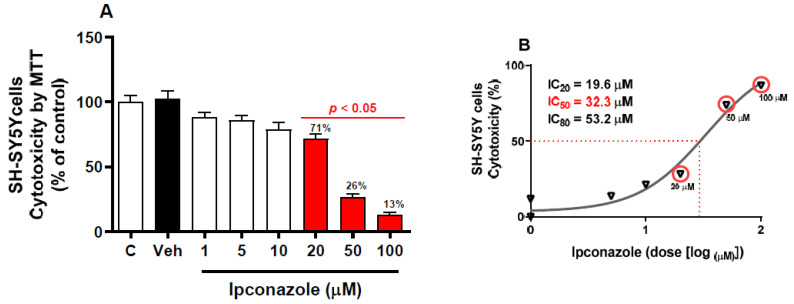
Cytotoxicity induced by ipconazole (1, 5, 10, 20, 50, and 100 µM) on SH-SY5Y cells after 24 h incubation period (**A**). Cytotoxicity was determined with MTT reduction, and a (%) dose–response curve was used to obtain the IC_20,50,80_ values (**B**). Data are presented as % control and as mean ± SEM of six repetitions. Ipconazole concentrations (red bars) show significant differences (*p* < 0.05) compared with the vehicle (Veh, black bar).

An MTT assay was performed to evaluate cell survival as a function of mitochondrial activity. There was no significant difference between the data from the vehicle group (0.1% DMSO) and the control group. The SH-SY5Y cells were exposed to different concentrations of ipconazole (1–100 µM) for 24 h (Figure 1). It was observed that ipconazole produced a significant (*p* < 0.05) dose-dependent cytotoxic effect, where cell viability decreased (compared with vehicle) with an increasing ipconazole dose (20 µM, 29%; 50 µM, 74%; and 100 µM, 87%) (Figure 1A). IC_20_ (19.6 µM), IC_50_ (32.3 µM), and IC_80_ (53.2 µM) values were also obtained for ipconazole (Figure 1B).

### 3.2. Effect of Ipconazole on ROS Production in SH-SY5Y Cells

**Figure 2:**
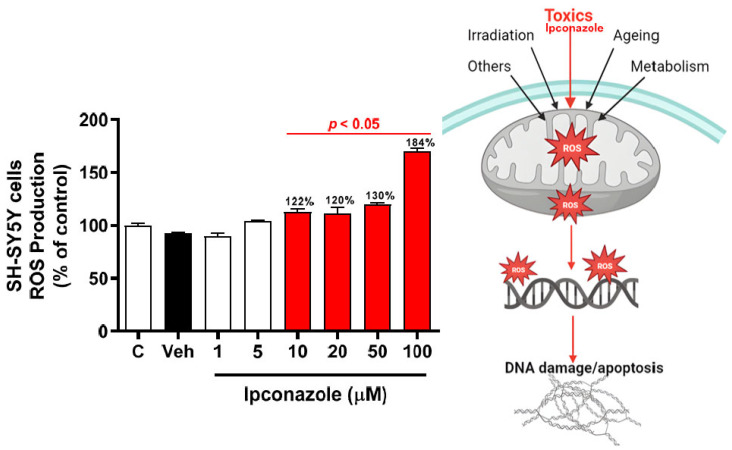
ROS production induced by ipconazole (1, 5, 10, 20, 50, and 100 µM) on SH-SY5Y cells after 24 h incubation period. ROS production was determined with DCFH-DA assay. Data are presented as fluorescence units and as mean ± SEM of six repetitions. Ipconazole concentrations (red bars: 10, 20, 50, and 100 µM) show significant differences (*p* < 0.05) compared with the vehicle (Veh, black bar). The figure on the right shows the possible mechanism of ROS production induced by toxic compounds such as ipconazole (adapted from [17]).

ROS production levels showed no significant difference between the vehicle group (DMSO 0.1%) and the control group. After a 24 h incubation period, ipconazole (10, 20, 50, and 100 µM) induced a significant increase of more than 20% (*p* < 0.05) in a dose-dependent manner in ROS production with respect to the vehicle group (Figure 2), whereas lower doses of ipconazole (1 and 5 µM) did not produce a significant increase in ROS production. Furthermore, the possible mechanism by which ipconazole could induce an increase in ROS production in SH-SY5Y cells can be observed in the figure on the right.

### 3.3. Effect of Ipconazole on Caspase3/7 Activity in SH-SY5Y Cells

**Figure 3:**
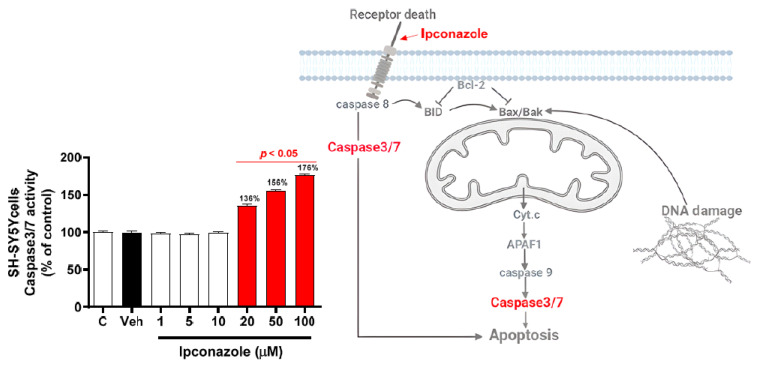
Caspase3/7 activity induced by ipconazole (1, 5, 10, 20, 50, and 100 µM) on SH-SY5Y cells after 24 h incubation period. Data are presented as % of control and as mean ± SEM of six repetitions. Ipconazole concentrations (red bars: 20, 50, and 100 µM) show significant differences (*p* < 0.05) compared with the vehicle (Veh, black bar). The figure on the right shows the possible mechanism of caspase 3/7 activity induced by ipconazole (adapted from [18]).

The enzymatic activity of caspase3/7 in SH-SY5Y cells was similar between the vehicle and control groups. Ipconazole at the concentrations of 20, 50, and 100 µM produced a significant (*p* < 0.05) increase in caspase3/7 activity, by 36%, 56%, and 76%, respectively, compared with the vehicle group (Figure 3). In addition, the possible mechanism by which ipconazole could induce an increase in caspase3/7 enzyme activity in SH-SY5Y cells can be seen in the figure on the right. These results confirm the association between the induction of oxidative stress (increased ROS production) and the induction of cell death (increased caspase3/7 activity).

### 3.4. Effect of Ipconazole on Cell-Death-Related Gene Expression in SH-SY5Y Cells

**Figure 4:**
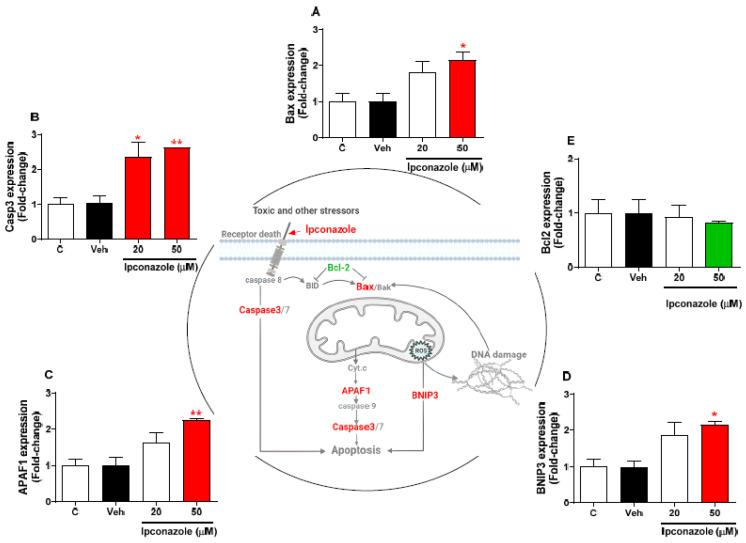
Effect of ipconazole (20 and 50 µM) after a 24 h incubation period on gene expressions of cell death mediators Bax (**A**), Casp3 (**B**), APAF1 (**C**), BNIP3 (**D**), and Bcl2 (**E**) in SH-SY5Y cells. Data represent the mean ± SEM of three repetitions. Up-regulated (red bar) and down-regulated (green bar) genes are significant (* *p* < 0.05 and ** *p* < 0.01) compared with the vehicle (black bar). The central figure shows the possible mechanism of cell death induced by ipconazole (adapted from [18]).

Expression profiling of cell-death-related genes by the effect of two concentrations of ipconazole (20 µM < IC_50_ > 50 µM) in SH-SY5Y cells was performed using real-time RT-PCR (Figure 4). In SH-SY5Y cells exposed to ipconazole (50 µM), the Bax gene was observed to be significantly increased, 2.15-fold, compared with the vehicle (Figure 4A). Furthermore, ipconazole significantly increased the Casp3 expression at the concentrations of 20 µM (2.36-fold) and 50 µM (2.62-fold) (Figure 4B). Ipconazole at the 50 µM dose significantly increased the APAF1 gene levels (2.26-fold) compared with the vehicle (Figure 4C). Likewise, BNIP3 gene expression was significantly increased (2.16-fold) with ipconazole (50 µM) compared with the vehicle (Figure 4D). However, ipconazole (50 µM) decreased the Bcl2 expression, but not significantly (Figure 4E). The central figure shows the possible mechanisms involved in ipconazole-induced cell death.

### 3.5. Effect of Ipconazole on the Expression of Inflammasome-Related Genes in SH-SY5Y Cells

**Figure 5:**
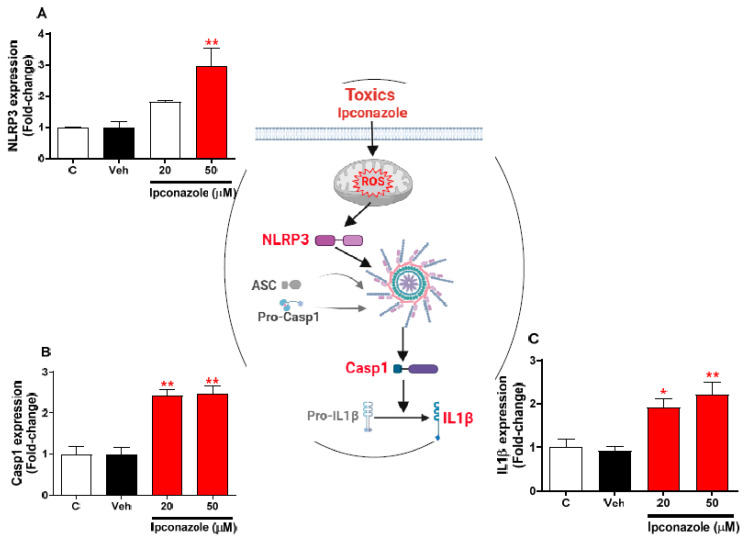
Effect of ipconazole (20 and 50 µM) after a 24 h incubation period on gene expressions of inflammasome mediators NLRP3 (**A**), Casp1 (**B**), and IL1β (**C**) in SH-SY5Y cells. Data represent the mean ± SEM of three repetitions. Up-regulated (red bar) genes are significant (* *p* < 0.05 and ** *p* < 0.01) compared with the vehicle (black bar). The central figure shows the possible mechanism of ipconazole-induced inflammasome activation (adapted from [19]).

Expression profiling of inflammasome-related genes with the effect of two concentrations of ipconazole (20 µM < IC_50_ > 50 µM) in SH-SY5Y cells was performed with real-time RT-PCR (Figure 5). In SH-SY5Y cells exposed to ipconazole (50 µM), the NLRP3 gene was observed to be significantly increased, 2.95-fold, compared with the vehicle (Figure 5A). Likewise, ipconazole significantly increased Casp1 and IL1β expression at the 20 µM (2.42- and 1.92-fold, respectively) and 50 µM (2.47- and 2.20-fold, respectively) concentrations (Figure 5B,C). The central figure shows the possible mechanisms involved in inflammasome activation by ipconazole.

### 3.6. Effect of Ipconazole on the Expression of Inflammation-Related Genes in SH-SY5Y Cells

**Figure 6:**
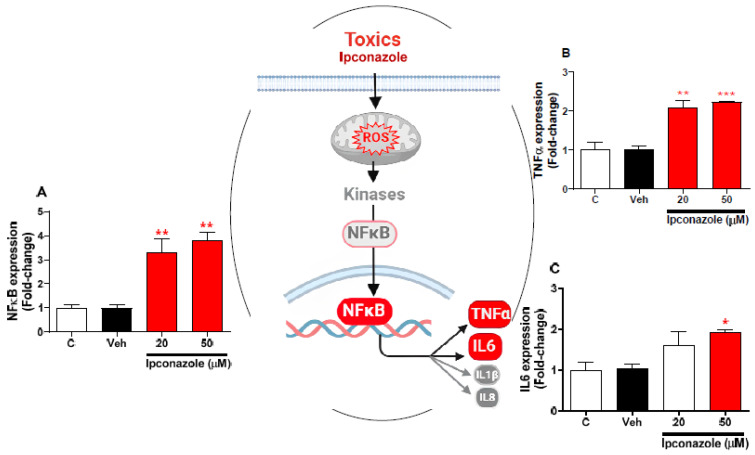
Effect of ipconazole (20 and 50 µM) after a 24 h incubation period on gene expressions of inflammation mediators NFκB (**A**), TNFα (**B**), and IL6 (**C**) in SH-SY5Y cells. Data represent the mean ± SEM of three repetitions. Up-regulated (red bar) genes are significant (* *p* < 0.05, ** *p* < 0.01, and *** *p* < 0.001) compared with the vehicle (black bar). The central figure shows the possible mechanism of ipconazole-induced inflammation (adapted from [20]).

Molecular expression of inflammation-related genes was evaluated with the effect of two concentrations of ipconazole (20 µM < IC_50_ > 50 µM) in SH-SY5Y cells (Figure 6). In SH-SY5Y cells, ipconazole significantly increased the NFκB and TNFα expression at the 20 µM (3.29- and 2.08-fold, respectively) and 50 µM (3.79- and 2.22-fold, respectively) concentrations compared with the vehicle (Figure 6A,B). Furthermore, ipconazole at 50 µM was observed to significantly up-regulate the IL6 (1.94-fold) gene compared with the vehicle (Figure 6C). The central figure shows the possible mechanisms involved in the activation of inflammasome regulators by ipconazole.

### 3.7. Effect of Ipconazole on the Expression of Antioxidant-Related Genes in SH-SY5Y Cells

**Figure 7:**
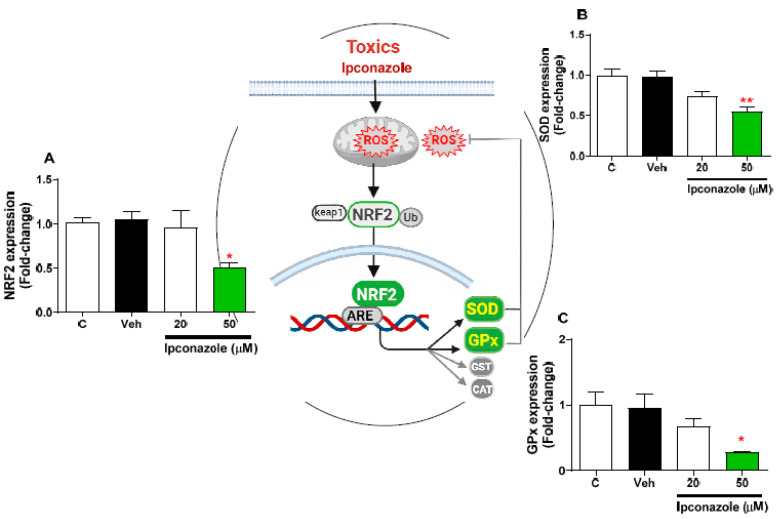
Effect of ipconazole (20 and 50 µM) after a 24 h incubation period on gene expressions of antioxidant mediators NRF2 (**A**), SOD (**B**), and GPx (**C**) in SH-SY5Y cells. Data represent the mean ± SEM of three repetitions. Down-regulated (green bar) genes are significant (* *p* < 0.05 and ** *p* < 0.01) compared with the vehicle (black bar). The central figure shows the possible mechanism of antioxidant mediators decreased by ipconazole (adapted from [21]).

Molecular expression of antioxidant-function mediator genes (NRF2, SOD, and GPx) was evaluated using the effect of two concentrations of ipconazole (20 µM < IC_50_ > 50 µM) in SH-SY5Y cells (Figure 7). In SH-SY5Y cells, ipconazole at the 50 µM concentration significantly decreased the expression of NRF2 (0.50-fold) (Figure 7A), SOD (0.56-fold) (Figure 7B), and GPx (0.28-fold) (Figure 7C) compared with the vehicle. The central figure shows the possible antioxidant mechanism altered by the effect of ipconazole.

## 4. Discussion

Triazole fungicides, such as ipconazole, are widely used in agriculture and livestock feed and can be ubiquitously detected in the environment. However, the human health and ecological risks associated with the presence of ipconazole are unclear. This study presents the neurotoxic effects of ipconazole exposure in SH-SY5Y cells, a cell line widely used in toxicological research.

Our results show that the fungicide ipconazole produced cytotoxic effects on human neuroblastoma SH-SY5Y cells (Figure 1). Cell viability assays (MTT reduction) showed an IC_50_ value of 32.3 µM for ipconazole, confirming the cytotoxicity of triazoles in cell cultures, as in the case of epoxiconazole, which reduced the viability of PC12 cells in a dose-dependent manner, with an IC_50_ of approximately 20 µM [22]. Moreover, the present study shows that exposure to ipconazole (10, 20, 50, and 100 µM) induces significant oxidative stress in SH-SY5Y cells, as was evident by the ROS production (22%, 20%, 30%, and 84%, respectively) (Figure 2). This result indicates a close relationship between increased ROS generation and exposure to triazole fungicides. ROSs are produced during mitochondrial oxidative metabolism and in response to cellular stress caused by xenobiotics (such as chemical compounds), cytokines, and biological agents [23]. Other studies in PC12 neuronal cells indicate that epoxiconazole causes elevated oxidative stress generation (increased intracellular ROS) [22] or that low concentrations of epoxiconazole (2, 5, and 10 µM) produce ROS generation in a dose-dependent manner in neuronal stem cells [24]. In fact, the nervous system is very sensitive to the effect of free radicals, due to its high rate of oxygen utilization. Excessive ROS production can cause strong and even irreversible damage to biomolecules such as proteins, lipids, and DNA [25], leading to cell failure, which could eventually lead to the induction of molecules such as caspases that promote neuronal cell death [26]. 

In the present study, caspase3/7 enzyme activity (Figure 3) and the molecular biomarkers Bax, Casp3, APAF1, and BNIP3 (Figure 4) were examined to confirm whether ipconazole was able to activate these molecules involved in cell death activation. After ipconazole exposure, the caspase3/7 enzyme was induced in a dose-dependent manner (20, 50, and 100 µM), and the molecular biomarkers Bax, Casp3, APAF1, and BNIP3 were mainly up-regulated with the 50 µM dose of ipconazole. These results indicate that ipconazole is a chemical agent involved in the activation of several cell death pathways. Indeed, activation of caspase enzymes is a key feature of apoptosis execution [27]. Thus, another triazole induced apoptosis in PC12 cells [22], in neural stem cells [24], and in kidneys of male rats [28] through caspase activation in a dose-dependent manner after 24 h of treatment. The caspase3/7 enzymes studied here generate apoptosis (programmed cell death) through different processes, with caspase3 being the main executioner of apoptotic death; caspase7, which plays no role in intrinsic sensitivity to apoptosis, may cause an accumulation of ROS that plays mainly a supportive role in the execution phase of apoptosis [29]. Likewise, oxidative stress and apoptosis are known to be closely related cellular processes in which, in addition to caspase enzymes, apoptotic mediators of the Bcl2 family (Bax and BNIP3) and APAF1 are involved [30]. Bax, which was up-regulated by ipconazole (50 µM) in this study, is known to cause permeabilization of the mitochondrial outer membrane, thus initiating the process of apoptosis [31]. On the other hand, caspase3 (up-regulated by ipconazole at 20 and 50 µM) acts downstream of Bax to control the execution of apoptosis. Thus, an increase in Bax expression results in increased activation of mitochondria-dependent capase3 enzyme and apoptotic cell death [32]. Similar apoptotic events were observed for other triazoles such as epoxiconazole [22,24] and tebuconazole [28]. It was also determined that ipconazole (50 µM) induced APAF1 overexpression in SH-SY5Y cells. APAF1 is a key molecule in the intrinsic apoptosis pathway that is activated in response to cytochrome c release and forms the apoptosome complex that triggers cell death. Intracellular stress signals, such as ROS generation, lead to the release of cytochrome c from mitochondria, which is mediated and regulated by the Bcl2 family [33,34], indicating the close relationship between the presence of ROS and the cell death biomolecules (caspases, Bax, BNIP3, and APAF1) evaluated in this study.

Cell death is a process involving different cellular pathways and different molecular activators such as the NLRP3 inflammasome complex, composed of the NLRP3 receptor protein, the adaptor protein ASC, and the Casp1 protease, which responds to different stimuli, such as environmental stimuli. The assembled NLRP3 inflammasome can activate Casp1 protease, induce apoptosis, and facilitate the release of IL1β and IL-18, triggering an exacerbated inflammatory response [35,36,37]. Our results show that the inflammasome complex was activated by the ipconazole effect in SH-SY5Y cells, as this fungicide was able to induce molecular up-regulation of NLRP3 (2.95-fold, at 50 µM ipconazole), Casp1 (2.42- and 2.47-fold at 20 and 50 µM ipconazole, respectively), and IL1β (1.92- and 2.20-fold at 20 and 50 µM ipconazole, respectively). In this study we have shown that ipconazole induces the generation of ROS that could be related to the gene overexpression of molecules of the inflammasome complex (NLRP3, Casp1, and IL1β). It has been proposed that ROS could be part of the signal for NLRP3 inflammasome activation [38,39,40] because it was found that ROS generated by dysfunctional mitochondria were able to activate the NLRP3 inflammasome in response to exogenous stimuli [41]. Exposure of nerve cells to pesticidal compounds such as ipconazole could be implicated in neurodegenerative processes, as studies have shown that the inflammasome complex is involved in the execution of inflammatory responses and pyroptotic death, leading to neurodegeneration. For this reason, it is possible that the inflammasome signaling pathway is a factor in the presentation of Alzheimer’s disease (AD), as increased IL1β was associated with the response to beta-amyloid (Aβ) deposition [42]. In this study, it was observed that IL1β was overexpressed by the effect of ipconazole, and in another study it was reported to be up-regulated in AD patients in Aβ-treated neurons and in transgenic AD mice [42,43,44,45]. Furthermore, it is suggested that Aβ, through activation of the NLRP3 complex, could be involved in the presentation of AD, as its activation in the microglia triggers neuroinflammation [45,46]. In this study, ipconazole resulted in the overexpression of molecules that compose the inflammasome pathway in SH-SY5Y cells, a well-established in vitro dopaminergic model for the study of Parkinson’s disease (PD). Thus, some studies suggest that activation of the NLRP3 inflammasome pathway is associated with the onset of PD [47,48]. Therefore, our findings suggest that ipconazole could be considered a factor that could lead to the development of neurodegenerative diseases.

Oxidative stress is the result of an imbalance due to excess ROS production or a weak or ineffective antioxidant response [23]. Neuroinflammation and oxidative stress are processes that in a prolonged form lead to neurodegeneration [49]. In this study, it was observed that ipconazole significantly increased the molecular expression of inflammation-related genes (NFκB, TNFα, and IL6) and reduced the molecular expression of antioxidant genes (NRF2, SOD, and GPx) in SH-SY5Y cells. Several studies have shown that various pesticides are capable of elevating levels of inflammatory factors, leading to neuroinflammation and impaired neuronal activity [50]. When the nervous system is exposed to pesticides [14,15,51,52], some nerve cells undergo activation of the NLRP3 inflammasome [53], including simultaneous activation of NFκB and other pathways, leading to the production and secretion of a large number of inflammatory factors such as IL1β, IL6, and TNFα, among others [54,55]; this increases mitochondrial dysfunction, with a consequent generation of ROS, MDA, and NO and a decrease in GSH and antioxidant enzymes such as CAT, SOD, and GPx [14,15,52], as we have reported in this study. Likewise, NRF2 (master of antioxidant regulation), which is activated by oxidative molecules such as ROS or by neurotransmission, needs to dissociate from the cytosolic protein KEAP1, so NRF2 enters the cell nucleus, resulting in the expression of several genes regulating antioxidant activity, including GPx, SOD, and others [21,56]. However, when neuronal homeostasis is altered and excessive ROS/RNS production is promoted, this could decrease NRF2 activity, and therefore also the synthesis and activity of the antioxidant system composed of SOD (which catalyzes the dismutation of superoxide anions into hydrogen peroxide) and GPx (which degrades hydrogen peroxide into water and oxygen) [56,57], enzymes molecularly evaluated in the present study and which were decreased by ipconazole.

## 5. Conclusions

Our work includes new data indicating that the induction of oxidative stress (ROS production) by ipconazole could be key to causing greater activity and expression of biomarkers related to cell death, as well as the activation of inflammasome complex factors, and that this could exacerbate a neuroinflammatory response or cell death in SH-SY5Y cells. Furthermore, this research provides a list of genes and signaling pathways that could be associated with neurotoxicity and neurodegeneration.

## Data Availability

Not applicable.
